# Experimenter evidence unmasking as a confound in optional stopping

**DOI:** 10.3758/s13428-025-02813-0

**Published:** 2025-09-24

**Authors:** Renata Sadibolova, Devin B. Terhune

**Affiliations:** 1 Department of Psychology, Institute of Psychiatry, Psychology & Neuroscience, King’s College London, London, UK; 2 School of Psychology, Roehampton University, London, UK

**Keywords:** Optional stopping, Experimenter blinding, Experimenter effects, Internal validity

## Abstract

Optional stopping refers to the practice of repeatedly performing a statistical analysis on a dataset as new data are collected until a pre-specified decision criterion is reached. This procedure is often adopted because of its effectiveness in optimizing data collection. Discussions of optional stopping to date have primarily centred around statistical issues, with relatively little consideration of any methodological implications of this procedure. Building on recent work drawing attention to methodological biases arising from the use of optional stopping, we highlight experimenter awareness of the current evidence state during data collection (*experimenter evidence unmasking*) as a salient methodological confound of optional stopping. We argue that experimenter evidence unmasking has the potential to influence an experimenter to implicitly or explicitly modify their behaviour in ways that can reduce the internal validity of an experiment. We conclude by offering recommendations for circumventing this confound and for the transparent reporting of experimenter evidence masking procedures.

## Optional stopping

Optional stopping refers to the methodological practice in empirical research where data collection is terminated based on the interim results in order to optimize data collection (Lakens, 2014; Rouder, 2014; Schonbrodt et al., 2017). This decision is typically based on a pre-defined stopping rule that specifies the criterion for stopping the experiment, such as the magnitude or stabilization of the effect size, the *p*-value, the Bayes factor, or the width of the credible interval. This procedure deviates from the more widely adopted stopping procedure of collecting data until a specific sample size is achieved as pre-determined by an *a priori* power analysis (O’Keefe, 2007). As such, optional stopping offers the potential to increase the efficiency of data collection and reduce the number of participants required for demonstrating an experimental effect.

Experimental researchers hold mixed views on the use of optional stopping. Optional stopping in the context of frequentist statistics (i.e., null hypothesis significance testing), such as by using *p* <.05 as a stopping criterion, is widely considered to reflect a questionable research practice because of type I error inflation (John et al., 2012; Nuijten et al., 2016; Simmons et al., 2011; Yu et al., 2014). However, it has been argued that this bias may be circumvented by using effect size stabilization as a stopping threshold (Anderson et al., 2021; Kowialiewski, 2024); for an extended discussion of approaches for mitigating bias in the analysis of sequential testing using null hypothesis significance testing, see Lakens (2014). By contrast, Bayesian optional stopping (also known as sequential Bayesian testing or sequential Bayes factors; Schonbrodt et al., 2017), wherein stopping decisions are made on the basis of Bayes factors rather than *p*-values, is becoming increasingly common owing in part to its robustness to familywise error rate confounds (Rouder, 2014; Schonbrodt et al., 2017). Nevertheless, there is continued statistical criticism regarding Bayesian variants of this procedure (Anderson et al., 2021; de Heide & Grunwald, 2021; Sanborn & Hills, 2014; Steele, 2013). A precise estimate of the prevalence of optional stopping in its various forms is not yet available (Fiedler & Schwarz, 2015; John et al., 2012).

Optional stopping may unfold as follows. Consider an example where a researcher, Mika, is conducting an experiment with a repeated-measures design comparing two conditions. Mika is initially masked to her data for the first phase of data collection (*masked phase*) and subsequently implements optional stopping by first performing a paired-samples *t*-test once she has reached a pre-specified default sample size (e.g., *n* = 20; Simmons et al., 2011). During the *unmasked phase* of data collection, she then sequentially performs this test as new data are collected (e.g., after each participant) until the results have met a specific decision criterion, such as a significant *p*-value (*p* <.05) or a Bayes factor (*BF*) reflecting moderate (or stronger) evidence for the null or alternative hypothesis (i.e., *BF* < 0.33 or *BF* > 3.0). This method is viewed as efficacious because it permits Mika to terminate data collection once her decision criterion is reached, say after collecting 26 participants, rather than continuing to unnecessarily collect data past this threshold, such as might happen had she had continued data collection until reaching a pre-determined sample size (e.g., *N* = 42) or discontinuation date (e.g., end of academic term). For this and other reasons, optional stopping, particularly when used with Bayesian statistics, has been widely endorsed and adopted in recent years (Rouder, 2014; Schonbrodt et al., 2017).

To date, discussions of the practice, utility, and potential concerns of optional stopping have centred around statistical biases that arise from this practice, such as type I error inflation (Anderson et al., 2021; de Heide & Grunwald, 2021; Rouder, 2014; Sanborn & Hills, 2014; Schnuerch et al., 2024; Schonbrodt et al., 2017; Tendeiro et al., 2021). By contrast, there has been relatively little attention to the possibility that optional stopping can introduce methodological biases (Beffara Bret et al., 2021; Elsey et al., 2021; Lakens, 2014). The possibility that optional stopping could introduce bias through a modification of experimenter behaviour was briefly introduced by Lakens (2014). A more expanded discussion of this effect, described as a form of interpersonal bias, along with a comprehensive set of solutions (see below), is provided by Beffara Bret et al. (2021). Similarly, Elsey et al. (2021) highlight the importance of ensuring that experimenters are blind to interim results, which they refer to as *insulated sequential testing*. They describe various scenarios under which an experimenter’s awareness of the current data could potentially impact their behaviour.

In this paper, we build upon this earlier work by introducing the term *experimenter evidence unmasking* as a methodological source of bias that parallels condition and group unmasking. We maintain that experimenter evidence unmasking can confound the internal and external validity of an experiment when optional stopping is employed. In contrast with statistical biases that have been argued to be specific to frequentist or Bayesian variants of optional stopping, we maintain that *all* forms of optional stopping are potentially susceptible to this confound, irrespective of the decision criterion. Although we focus on optional stopping, this confound will also be present in studies that do not formally adopt optional stopping but in which the experimenter is unmasked to their data. Accordingly, experimenter evidence unmasking should be regarded as a broad source of potential bias that spans all statistical approaches and stopping rules. In what follows, we outline how experimenter evidence unmasking can pose a threat to the validity of an experiment and describe how the magnitude of this confound will plausibly vary depending on the evidence state. Optional stopping has the potential to introduce experimenter effects in the study of human behaviour with potential deleterious effects on the validity and reproducibility of experimental research. We describe remedies to this confound, such as automated data analysis (e.g., Beffara Bret et al., 2021) but argue that optional stopping possesses an inherent confound that can emerge in certain scenarios that cannot be circumvented through automation. Finally, we advocate for the transparent reporting of optional stopping and the specification of masked and unmasked phases of data collection so that the potential bias introduced by optional stopping can be quantified.

## Experimenter unmasking

*Experimenter masking* (or blinding) refers to the methodological practice of ensuring that an experimenter is unaware of some salient information pertaining to an experiment (Klein et al., 2012). Typically, it refers to blinding an experimenter to the condition to which a participant has been assigned to (e.g., placebo versus intervention) or of which group a specific participant is a member (e.g., control versus patient). In a conceptually similar fashion to the use of participant masking to minimize the impact of participants’ beliefs, expectations, and biases on experimental outcomes (Schulz & Grimes, 2002), experimenter masking is employed in numerous (quasi-)experimental designs in order to minimize *experimenter effects* (Holman et al., 2015). Experimenter effects refer to cases when an experimenter implicitly or explicitly influences a participant’s behaviour in a manner that is independent of the experimental manipulation or independent variable under study (Barber, 1976; Rosenthal, 1966). The introduction of a second source of causal influence over participants’ behaviour can reduce the internal validity of an experiment and compromise an experiment’s potential for properly estimating the effect size and robustness of the experimental manipulation.

Experimenter condition unmasking is a long-recognized confound in experimental designs in the study of human behaviour (Barber, 1976; Orne, 1962; Rosenthal, 1966). Nevertheless, despite decades of recognition of the impact of experimenter effects (Holman et al., 2015; Klein et al., 2012), the inclusion of unmasked experimenters remains highly prevalent in these fields (Scott et al., 2022) and may be even less recognized by contemporary researchers than their predecessors (Klein et al., 2012). In accordance with long-held concerns about the detrimental effects of unmasking, experimenter unmasking appears to inflate effect sizes: previous studies and meta-analyses suggest that the use of unmasked experimenters is associated with a 22–68% increase in effect sizes relative to the use of masked experimenters (Holman et al., 2015; Hrobjartsson et al., 2012, 2013, 2014; Savovic et al., 2012). Experimenter behaviour may further lead participants to lose their condition mask (blind), which may lead to the introduction of additional biases driven by participants’ expectations (Schulz & Grimes, 2002). Although it has been hypothesized that experimenter effects are more likely to occur in social psychological research (Klein et al., 2012), they can be observed in a variety of behavioural paradigms including in the assessment of non-human animal behaviour (Bohlen et al., 2014; Lit et al., 2011). Relatedly, insofar as verbal suggestions and placebo/nocebo manipulations are known to modulate neurophysiological responses (Colloca & Barsky, 2020; Oakley & Halligan, 2013; Terhune et al., 2017; Wager & Atlas, 2015), it is likely that experimenter effects are operating in many subdisciplines related to behaviour. Moreover, it should be noted that experimenter unmasking can yield effect size augmentation or attenuation, depending on experimenters’ theoretical orientations, as might be the case in adversarial collaborations (Cowan et al., 2020).

Experimenter unmasking should be understood as a threat to the internal validity of an experiment, as it introduces an additional variable (experimenter behaviour) that is distinct from the experimental manipulation under assessment. In turn, it has the potential to shape outcomes independently of, or in interaction with, the respective manipulation, and thereby can substantially hinder mechanistic research by biasing the estimation of experimental outcomes. In effect, experimenter unmasking amounts to introducing another source of variance in participant responses and thereby can mask the true effect size of an experimental manipulation or group difference.

## Experimenter evidence unmasking as a confound in optional stopping

A similar, albeit less frequently considered, confound is an experimenter’s awareness of the current evidence state during data collection, what we here call *experimenter evidence unmasking*. Experimenter evidence masking is the practice of ensuring that experimenters are unaware of the current (and past) evidence state until data collection is complete. We argue that experimenter evidence unmasking can function as a confound in experimental research by altering experimenter–participant interactions or by experimenters implicitly or explicitly modifying ongoing experimental protocols, thereby reducing standardization and, concomitantly, internal validity and reproducibility (Elson, 2019). Outside of the specific context of experimenter effects, the use of masked data analysts continues to grow in a variety of fields allied to the study of human behaviour, in recognition of the potential for unmasked data analysts to introduce bias in data analysis (Dutilh et al., 2021). Experimenter evidence unmasking may also facilitate questionable data analysis practices such as *p*-hacking (Wicherts et al., 2016). By contrast, the specific confound of experimenter evidence unmasking in regard to an experimenter’s behaviour has received relatively little attention (Beffara Bret et al., 2021; Elsey et al., 2021; Lakens, 2014) even though there is some preliminary evidence that evidence unmasking can impact experimental behaviour (Rosenthal, 2002).

Our central thesis is that experimenter evidence unmasking in the context of optional stopping has the potential to confound data collection in a manner that can threaten the internal validity of an experiment. In particular, if an experimenter becomes aware of statistical outcomes during data collection, this will generate belief states regarding the current evidence state. The evidence state can function as a form of feedback that strengthens or weakens belief states regarding whether an experimental manipulation is “working” or not and thus has the power to influence an experimenter’s behaviour. Irrespective of the accuracy of these belief states, they have the potential to lead experimenters to implicitly or explicitly modify their behaviour in the context of the study. These changes in an experimenter’s behaviour are likely to deviate from the experimental protocol, reducing standardization and thereby internal validity (Elson, 2019), similar to how knowledge of the condition to which a participant has been allocated (e.g., placebo vs intervention) can influence an experimenter’s interactions with a participant and introduce bias (Holman et al., 2015).

The influence of experimenter evidence unmasking is likely to depend on the nature of the respective evidence. Although the potential attenuating effect of optional stopping on internal validity applies irrespective of the statistical approach adopted, here we use the example of Bayesian optional stopping due to its growing popularity (Rouder, 2014; Schonbrodt et al., 2017). It is important to distinguish different features of Bayesian evidence that can be inferred from *BF*s during optional stopping at a specific timepoint during data collection: the magnitude, the direction, and the trajectory.

**Fig. 1:**
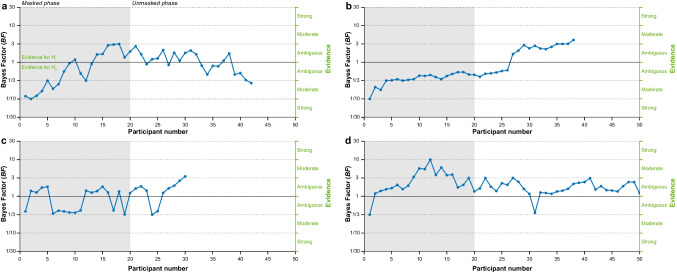
Simulated data demonstrating trajectories of Bayes factors (*BF*s) as a function of data collection stage (sequential participant number). All panels show *BF*s from *t*-tests including each new participant. In each case, it is assumed that the experimenter becomes unmasked at *n* = 20; grey and white regions denote experimenter masked and unmasked phases, respectively. *BF*s greater than and smaller than 1 denote evidence for the alternative (*H*_1_) and null (*H*_0_) hypothesis, respectively, with *BF*s greater than 3.0 and smaller than 1/3 indicating moderate to strong evidence for the *H*_1_ and *H*_0_, respectively. *BF* trajectories finish (i.e., data collection stops) when 0.33 > *BF* > 3.0

Within Bayesian optional stopping, the Bayes factor magnitude provides an estimate of the current evidential support for one model over another and directly informs decisions regarding whether to cease or continue data collection relative to a decision threshold pertaining to the evidence (i.e., ambiguous vs moderate-or-higher evidence). By convention, this threshold is often set at a Bayes factor of 3 in favour of the alternative *H*_1_ (*BF* > 3.0) or 1/3 in favour of the null *H*_0_ (*BF* < 0.33) hypothesis, reflecting moderate (or greater) evidence based on the relative marginal likelihoods of the data under the competing hypotheses (Jeffreys, 1961; Wagenmakers et al., 2018). It follows that the evidence will be considered insensitive until the respective pre-specified criterion is reached (Fig. 1). Nevertheless, an ambiguous Bayes factor (e.g., 0.33 < *BF* < 3.0) can still be salient to an experimenter. For example, a Bayes factor might be near to (e.g., *BF* = 2.8) or far from (e.g., *BF* = 1.2) the stopping threshold, and this provides the experimenter with information from which they can infer, incorrectly (Fig. 1a, n = 21) or correctly (Fig. 1b, n = 30), whether an experimental manipulation is likely to be effective or not. The Bayes factor direction, by contrast, provides information regarding whether the current evidence state is in favour of the null (*BF* < 1.0) or the alternative (*BF* > 1.0) hypothesis. For example, an experimenter may reasonably expect to observe *eventual* support for the alternative *H*_1_ if the majority of *BF* values are consistently larger than 1 (Fig. 1a, d). This similarly provides information that an experimenter might rely on to infer whether a manipulation is effective or not.

Finally, the Bayes factor trajectory provides information regarding its recent time course, variability, or slope during data collection; this information can impact an experimenter’s *subjective* estimates of how the evidence has changed or not and might be interpreted by an experimenter as an indication of the evidence state moving toward, or away from, or remaining ambiguous with respect to, the decision criterion. Again, inferences based on evidence trajectories may be accurate (Fig. 1b, c) or inaccurate (Fig. 1a, d), and they may interact with experimenter’s expectations for the results in shaping unmasking biases (Beffara Bret et al., 2021).

If an experimenter were to learn any of these pieces of information, it is plausible that this could shape their behaviour. Problematically, the impact of experimenter evidence unmasking is likely to differentially impact the experimenter’s behaviour depending on the evidence state, such as whether the data are in favour of the null or the alternative hypothesis. Accordingly, the influence of experimenter evidence unmasking will differ across contexts and exert a more pronounced influence in certain scenarios. Moreover, the impact of this bias may be highly dynamic and vary stochastically based on fluctuations in the evidence state over time. One of the significant consequences is that experimenter evidence unmasking is likely to exert a greater influence on data characterized by ambiguous evidence and thus research studies with less reliable, or weaker, effect sizes. These effects will also plausibly interact with an experimenter’s preferred hypothesis as well as condition/group unmasking, thereby potentially further augmenting experimental bias.

For example, consider a set of scenarios when Mika runs her analysis after collecting data from her first 20 participants (Simmons et al., 2011) and then continues to re-compute Bayes factors (unmasked) after each participant’s data are collected (see also Lakens, 2014). In one scenario (Fig. 1a), the Bayes factor is near to her pre-specified decision threshold for rejecting the null hypothesis (*BF* = 2.8; *n* = 21) and in favour of her preferred hypothesis. The evidence here signifies to Mika that her hypothesized effect is *ostensibly* present and is likely to function as explicit positive feedback (her manipulation seems to be effective, and she need *not* adjust her experimental behaviour). In this scenario, it is plausible that Mika will continue data collection according to her protocol and that experimenter evidence unmasking will not significantly threaten the internal validity of her experiment. Nevertheless, suspecting that she has nearly completed data collection, Mika may subtly modify how she interacts with the next participants, such as by devoting extra time to explaining the instructions or giving participants subtle clues, so as not to disrupt the apparent effect. Moreover, if she has access to participants’ real-time responses, she may treat differently those who show the response pattern not conforming to her hypothesis.

By contrast, in another scenario (Fig. 1b), the Bayes factor remains ambiguous at *n* = 26 and thus is not yet suggestive or informative. In addition, the evidence aligns more with the null hypothesis, despite constituting insensitive evidence. This circumstance may function as a source of disappointment or fatigue, as it may lead Mika to believe that she will need to collect data for a considerably longer period of time, that her hypothesized effect is not particularly robust, and/or that she might even have to discontinue data collection with an ambiguous effect (e.g., if she pre-specified to discontinue data collection in the event of an ambiguous evidence state at *N* = 50). These various factors have the potential to cause Mika to implicitly or explicitly modify her experimental behaviour in order to bolster this apparently weak effect, representing a clear threat to internal validity, due to a change in her experimental behaviour and deviation from the experimental protocol (Elson, 2019) relative to the pre-unmasking phase.

In our third and fourth simulated scenarios, the Bayes factor is highly variable but on an initial downward trend (Fig. 1c) and does not pass the decision threshold (Fig. 1d). Observing this *suggestive* downward linear trend (Fig. 1c) or observing a highly variable effect (Fig. 1d) may lead Mika to worry about the robustness of her hypothesized effect, prompting her again to modify her behaviour. She may respond by omitting certain instructions while emphasizing others and giving explicit verbal suggestions regarding performance or proffering particular strategies that participants should adopt. All of these changes represent potential threats to the internal validity of Mika’s experiment, particularly if the true effect aligns with the null hypothesis (Fig. 1a) or is weak or highly variable in magnitude (Fig. 1d).

These hypothetical scenarios illustrate how the current evidence state may influence an experimenter’s interactions with participants. Critically, the impact of the evidence state on experimental behaviour is likely to differ on the basis of the experimenter’s preferred hypothesis, as well as the magnitude, direction, and trajectory of Bayes factors. These scenarios demonstrate how experimenter evidence unmasking has a clear potential to reduce the internal validity of experiments of human behaviour and thus should be avoided.

## Avoiding experimenter unmasking

It should be clear that despite the value of various methods to minimize the potential for questionable research practices (John et al., 2012) such as preregistration (Nosek et al., 2018), registered reports (Chambers & Tzavella, 2021; Nosek & Lakens, 2014), or multiple data analysts (Wagenmakers et al., 2021), these practices are insufficient to address the confound posed by optional stopping, because it represents a methodological, rather than statistical, confound. Moreover, this confound will be present irrespective of the method of optional stopping (e.g., *p*-values vs Bayes factors) because an unmasked experimenter could still introduce biases during data collection given their knowledge of any type of evidence.

Nevertheless, the most pronounced threats to internal validity presented by experimenter evidence unmasking can be avoided without losing the potential benefits of optional stopping. The most salient way to mitigate the potential confounding effects of experimenter evidence unmasking is by minimizing or eliminating experimenter–participant interactions, such as through automation in the administration of instructions and tasks as in online experiments. However, even in experiments that require substantial participant–experimenter interactions, this confound can be avoided through the inclusion of masked data analysts, automated data analysis that maintains experimenter evidence masking (Beffara Bret et al., 2021; Elsey et al., 2021), or joint evidence and analysis design masking. In the first, a data analyst, also masked to experimental conditions, will perform the respective analyses and inform the experimenter whether data collection should cease or continue (Lakens, 2014). In order to completely preserve the internal validity of the study, this communication should be binary (“stop” or “continue”) and should not provide any information regarding the evidence state (magnitude, direction, or trajectory of statistical evidence). Alternatively, as recommended elsewhere to avoid experimenter effects (Beffara Bret et al., 2021; Hicks et al., 2006), data analysis could be fully automated and provide the experimenter a similar binary instruction without any information regarding the evidence state. To this end, Beffara Bret et al. (2021) introduced a fully automated, triple-blind protocol for implementing sequential testing using *R* with an easy-to-follow tutorial. Finally, the experimenter responsible for data collection could be jointly masked to both the evidence state and analysis *design*, such that they are completely unaware of both the interim results and the stopping rule (e.g., optional stopping vs pre-determined sample size). These measures will ensure that experimenters can reap the benefits of optional stopping (Rouder, 2014; Schonbrodt et al., 2017) whilst minimizing threats to internal validity incurred by experimenter evidence unmasking.

## An inherent methodological confound of optional stopping

Although we have described how the internal validity threats engendered by optional stopping can be mitigated, one feature of the evidence state is difficult to avoid when optional stopping is employed. Even if experimenter evidence masking is employed to a rigorous level (i.e., the experimenter is unaware of the current evidence state), an experimenter’s awareness that the data have not yet passed the decision threshold itself represents a potential threat to internal validity because it provides information about the evidence state (see also Elsey et al., 2021). That is, an experimenter is able to make inferences about the effect size, and/or its variance, of the manipulation as data collection proceeds. For example, if data collection has continued to *N* = 60 without passing the decision threshold, an experimenter is likely to infer that the effect size is either weak or characterized by high variance. Among the methods for mitigating the confounding effects of unmasking described above, joint experimenter evidence and analysis design masking is arguably the only one that could eliminate this confound, but it is likely to be challenging to practically implement in a rigorous manner. Nevertheless, it should be acknowledged that this information is less informative than knowing detailed specific statistical evidence (e.g., an effect size or Bayes factor). Accordingly, this form of experimenter evidence unmasking is likely to engender relatively weak confounding effects. Moreover, the confounding effect of unmasking in such cases is plausibly less pronounced when Bayesian optional stopping is used relative to frequentist variants, given that there are two possible stopping thresholds corresponding to the null and alternative hypotheses. Finally, it should be noted that this lingering inherent confound of optional stopping will be specific to experiments involving weak or variable effect sizes. This highlights the difficulty in completely avoiding the confound of experimenter evidence unmasking in participant-facing contexts with single experimenters and thus represents an inherent methodological flaw of optional stopping that warrants greater consideration.

## Quantifying the presence and magnitude of experimenter evidence unmasking bias

At present, the prevalence of optional stopping and experimenter evidence unmasking, as well as the magnitude of any bias in data collection driven by the latter, is unknown (Elsey et al., 2021). The potential impact of optional stopping in individual experiments can be quantified and statistically assessed by contrasting data prior to (masked phase) and after the introduction of optional stopping (unmasked phase) (Fig. 1). Researchers, in turn, can report an effect size for this phase difference and associated measure of uncertainty (e.g., confidence or credible intervals) as a quantitative estimate of potential bias introduced by experimenter evidence unmasking. As always, researchers should be cautious in interpreting such effect sizes and be mindful about low statistical power and overinterpreting non-significant or ambiguous effects. We recommend that researchers report this information in order to inform readers of the potential for bias due to the use of optional stopping as well as to move the field forward in understanding the magnitude and potential biasing effects of experimenter evidence unmasking.

## Transparent reporting of experimenter (un)masking

The use of optional stopping should be guided by clear guidelines and standards (Beffara Bret et al., 2021; Elsey et al., 2021). In particular, researchers should pre-register their stopping rules, report their methods transparently, and carefully consider the methodological implications of optional stopping. At present, experimenter (un)masking is widely underreported (Burghardt et al., 2012; Holman et al., 2015; Klein et al., 2012), thereby presenting a significant challenge in estimating the prevalence of the biases presented here as well as their potential impact (Lakens, 2014). Moreover, despite increased recognition of the importance of transparency at all stages of the research process, recent papers offering guidelines for research transparency (Wagenmakers et al., 2021) and the reporting of Bayesian statistics (Kruschke, 2021) have not made reference to experimenter evidence unmasking. The recent *transparency checklist* (Aczel et al., 2020) includes a partial reference to it, encouraging authors to report “whether (and, if so, how) participants, experimenters, and data-analysts were kept naive to potentially biasing information”. However, many researchers will be unaware of experimenter evidence unmasking as a potential source of bias, and thus we recommend that such questions include greater specificity in future revisions and more detailed information regarding specific practices as well as masked and unmasked phases.

We recommend that all studies using optional stopping transparently report whether experimenter evidence masking was implemented or not, the sample size at which point the experimenter became unmasked, how masking was evaluated (Fassi & Cohen Kadosh, 2021), and how data were analysed, including the use of automated or masked analysts (Beffara Bret et al., 2021). This will be valuable in informing researchers and stakeholders about potential methodological sources of bias in an experiment. In turn, the presence or absence of such biases can then be considered when weighing the relative evidence for or against a specific hypothesis (Klein et al., 2012) and the methodological rigour of the respective study in systematic reviews and meta-analyses (Higgins & Green, 2008). Again, we further recommend that authors quantify the magnitude of potential bias by comparing unmasked and masked phases of data collection. Cumulatively, this transparency will also help to inform future empirical research on the extent to which experimenter evidence unmasking moderates effect sizes, as observed with experimenter condition unmasking (Holman et al., 2015), and whether this is specific to particular experimental paradigms.

## Summary and conclusions

With the potentially widespread use of optional stopping to optimize data collection in the behavioural sciences (Lakens, 2014), greater consideration of methodological limitations of this procedure are warranted. Here we present experimenter evidence unmasking as an underappreciated methodological confound of optional stopping that has the potential to function as a threat to the internal validity of experiments in which this procedure is adopted. We outline different scenarios by which this confound may bias data collection and highlight remedies for circumventing it (Beffara Bret et al., 2021; Elsey et al., 2021) whilst acknowledging lingering problematic features of this procedure. We hope that by drawing greater attention to this confound, we will bring increased recognition to methodological and statistical confounds arising from experimenter unmasking in its various forms.

## Data Availability

https://osf.io/nr5ht/

## Code availability

https://osf.io/nr5ht/

## Declarations

### Open practices statement

The code for computing Bayes factors and plotting Bayes factor trajectories is available on the Open Science Framework: https://osf.io/nr5ht/

### Open access

For the purpose of open access, the author has applied a CC BY public copyright licence (where permitted by UKRI, an Open Government Licence or CC BY ND public copyright licence may be used instead) to any Author Accepted Manuscript version arising.
